# Wayfinding: How Ecological Perspectives of Navigating Dynamic Environments Can Enrich Our Understanding of the Learner and the Learning Process in Sport

**DOI:** 10.1186/s40798-020-00280-9

**Published:** 2020-10-28

**Authors:** Carl T. Woods, James Rudd, Sam Robertson, Keith Davids

**Affiliations:** 1 Institute for Health and Sport, Victoria University, Melbourne, Australia; 2 Research Institute for Sport and Exercise Sciences, Liverpool John Moores University, Liverpool, UK; 3 Sport & Human Performance Research Group, Sheffield Hallam University, Sheffield, UK

**Keywords:** Ecological dynamics, Wayfinding, Affordance landscape, Learning design, Perception-action coupling, Knowledge of/about

## Abstract

Wayfinding is the process of embarking upon a purposeful, intentional, and self-regulated journey that takes an individual from an intended region in one landscape to another. This process is facilitated through an individual’s capacity to utilise temporally structured, functional actions embedded within a particular environmental niche. Thus, individuals learn of their performance landscapes by experiencing them through interactions, detecting and exploiting its many features to ‘find their way’. In this opinion piece, we argue that these ecological and anthropological conceptualisations of human navigation can, metaphorically, deepen our understanding of the learner and the learning process in sport, viewed through the lens of ecological dynamics. Specifically, we consider sports practitioners as (learning) landscape designers, and learners as wayfinders; individuals who learn to skilfully self-regulate through uncharted fields (composed of emergent problems) within performance landscapes through a deeply embodied and embedded perception-action coupling. We contend that, through this re-configuration of the learner and the learning process in sport, practitioners may better enact learning designs that afford learners exploratory freedoms, learning to perceive and utilise available opportunities for action to skilfully navigate through emergent performance-related problems. We conclude the paper by offering two practical examples in which practitioners have designed practice landscapes that situate learners as wayfinders and the learning process in sport as wayfinding.

## Key Points

- Theoretically positioned in an ecological dynamics framework, this paper re-conceptualises learners in sport as wayfinders and the subsequent performance and learning process as wayfinding.
- Through this re-configuration, sports practitioners may better enact learning designs that afford learners opportunities to search and explore performance landscapes in practice, learning to perceive and utilise available opportunities for action, empowering them to actively self-regulate through emergent, performance-related problems.
- Two examples from across the sporting landscape are presented, bringing life to this proposal of re-conceptualising the performance and learning process.

## Introduction

*“… wayfinding is an activity that confronts us with the marvellous fact of being in the world, requiring us to look up and take notice, to cognitively and emotionally interact with our surroundings”* – M.R. O’Connor

Ecological dynamics is a contemporary, transdisciplinary theoretical framework on learning and performance [1], which integrates concepts from ecological psychology [2], constraints on dynamical systems [3, 4], the complexity sciences [5] and evolutionary science [6]. It invites the re-conceptualisation of the work of support practitioners in sport, physical activity and education, through advocating the mutuality of the individual and performance environment [7, 8]. Within this framework, related concepts such as skilled behaviour, learning, expertise and talent, are viewed as emergent properties of a functionally adaptable, evolving relationship formed between a performer and the constraints of his/her environment [9]. Skilful actions are not viewed as repetitious movements of the body removed from context, but are dynamic, body-environment interactions [10], through which individuals self-regulate to achieve their intended task goals.

In this ecological framework, the necessary process of self-regulation is supported by the development and exploitation of deeply entangled relationships between an individual’s perceptions, actions, cognitions, emotions and the dynamics of a performance environment. The individual and environment are viewed as being mutually reciprocal [2], since information in the environment shapes an individual’s actions and vice versa [11]. Learning, framed within this ontology, can be understood as a process by which an individual is empowered to progressively deepen knowledge of the environment and his/her place within it, exploring how action capabilities can be adapted to suffice an ever-evolving array of constraints [9]. Accordingly, skill acquisition has been repositioned as skill ‘adaptation’ within an ecological dynamics framework [9].

In sport, practitioners (e.g. trainers, teachers, coaches, sport scientists and analysts) working within this framework are challenged to re-configure their role in performance preparation, evolving toward the designer of landscapes that learners (e.g. students and athletes) can interact with, not the conveyers of declarative knowledge about how a problem ‘should’ be solved. If practice conditions are designed appropriately, learners will actively self-regulate their interactions with a specifically designed practice environment to discover how to best achieve an intended task outcome, based on their current action capabilities [12]. Here, we contend that self-regulation is better understood as an ‘active’ process, where, through careful task manipulations and informed practice designs, a practitioner works with a learner to guide, direct or nudge (when appropriate) his/her attention toward specific features of his/her environment of use for exploiting actions.

In this opinion piece, we argue that these ecological conceptualisations in ecological dynamics not only re-define the role of support practitioners in performance preparation, but challenge us to re-configure our understanding of the learner and the learning process. To guide this re-configuration, we consider practitioners as designers and learners as wayfinders; metaphorically situated as individuals who skilfully navigate through uncharted fields (i.e., performance-related problems) within a landscape (i.e., competition or practice tasks), supported by a deeply entangled relationship between perception, cognition, emotion and action. As we will argue, wayfinding is not an inherently novel concept [10, 13–15], but one with connotations applicable for understanding learning and skilled action in sport through an ecological dynamics lens. By re-configuring our understanding of the learning process, we may better enact learning designs that afford learners the freedom to explore and self-regulate through uncharted fields of their emerging performance landscapes.

### Wayfinding and Self-Regulation

#### More Than Just Navigating Across Fixed Points in Space

In its literal sense, wayfinding is viewed as a purposeful, intentional and self-regulated movement that takes an individual from one region in a landscape to another [14]. As argued by the prominent social anthropologist, Tim Ingold [10], wayfinding should not be understood though, as simply navigating between fixed points in space. Such a superficial insight, according to Ingold [10], is akin to *transport*, where an individual is more interested in reaching a pre-planned destination by transiting ‘across’ a landscape, as opposed to moving ‘through’ a landscape. This distinction is important for our paper, as it emphasises that an individual learns of their landscape through interactions as they move *through* it, not by skimming *across* it, developing a deep, embedded and evolving individual-environment relationship as they go. Stated more apparently, it is the ‘journey’ that is of relevance to a wayfinder, not just the arrival at a ‘destination’ [10].

In his book, The Perception of the Environment, Ingold assimilates these ideas on wayfinding to music playing, where a musician exemplifies wayfinding by emotionally engaging with the music’s beat and tempo (viewed as informational constraints within ecological dynamics), adjusting his/her playing to ‘fit’ within the broader orchestra of sounds. The particular ‘path’ being navigated by the improvising musician is in the playing of music that unfolds, and the emerging ‘vistas’ they traverse are encompassed within the song’s beat and tempo. Indeed, this interpretation of wayfinding does detach from its literal meaning, as a music ensemble is not physically traversing through different regions in a landscape. However, while metaphoric, a musician does attend to emerging information during the song that enables their continued improvisation of sound and timing to successfully ‘find their way’ through the sonic ‘landscape’ being created. It is this underlying and dynamic *process*, captured in the interaction between the musician and music (performer and environment), that helps them find their way through the song. Here, we base our interpretations of wayfinding in a similar lens to that of Ingold [10]. We acknowledge that this interpretative exercise does somewhat detach ‘wayfinding’ from its more literal connotation. However, we argue that this re-conceptualisation still preserves the underlying, and dynamic processes of wayfinding when applied to sport and predicated on an ecological dynamics rationale. Specifically, it is proposed that the competitive performance landscape in sport is constantly evolving and undulating, and athletes must subsequently learn to wayfind through these landscapes by adapting their performance behaviours to emergent constraints. This ecological dynamics interpretation of wayfinding, we argue, is valuable for understanding the process of athlete self-regulation in a dynamically changing competitive performance landscape, which evolves over the relevant timescale of sport performance [1].

Re-conceptualising the learning process in sport through such a dynamical lens would require an individual to develop intimate knowledge of a landscape’s informational constraints such as physical features, climate, socio-cultural norms, rules and local history, understanding how such things enmesh to shape his/her perceptions and actions through an evolving “long-term attunement and attentiveness” ([16], p. 225) to various opportunities for environmental interaction. Thus, we contend and seek to exemplify throughout this paper, that sporting competitions or activities could be metaphorically understood as performance ‘landscapes’. The emergent and decaying problems and challenges are subsequently represented in the many ‘fields’ that athletes and students learn to navigate through, not by following a path pre-defined by a practitioner, but by progressively learning to become responsive to the available opportunities for action—thereby exemplifying wayfinding.

#### Ecological Perspectives of Wayfinding

Indeed, ecological perspectives of this rationale have been shaped by psychologist James Gibson [2, 17] in his rejection of the inherent, traditionally dualistic belief that humans possessed ‘cognitive maps’ in their brains of use in navigating the world. He argued that there was no separation, now termed *organismic asymmetry* [18], between the mind and environment or between perceiving and knowing, and that wayfinding exemplified the real-time coupling and embodiment between perception, cognition and action. He proposed that individuals navigating performance landscapes relied upon temporally structured actions specifically entwined within a particular environmental niche, not internal representations or ‘cognitive maps’ stored in their memory [10, 17]. Given the vastness of distances between regions, preventing perception of the whole environmental layout from a single vantagepoint, he argued that individuals learned to navigate regions within a landscape by *experiencing* them.

The origins of this definition are subsequently grounded in ecological perspectives of how humans navigated the world without modern day technological devices, such as compasses or Global Positioning Systems (GPS). At this point, we link wayfinding to the learning process in sporting environments by encouraging the reader to metaphorically consider the more traditional, mechanistic and autocratic teaching or coaching pedagogies in a similar vein to a compass or GPS device—that is, modes of explicit knowledge conveyance, from an external source (i.e., instructor, trainer, coach or parent), about how learners should perform (and repeat) some idealistic movement template (i.e., following the ‘fastest’ destination route as selected by a GPS device to get to a fixed location in space). These ideas imply how the sports practitioner, who determines a learner’s interactions with a performance environment from such a global-to-local direction [19], acting somewhat like a compass or GPS device for a learner, is likely to hinder an individual’s capacities to experience the environment by interacting with it (in a local-to-global direction [19]), reducing their capacity to self-regulate through it [20]. To consolidate this point, we ask readers to consider the last time they utilised a GPS device for navigational purposes—how attuned or responsive were you to the subtleties of your environment that could be used to inform your navigation (i.e., features ‘outside’ of the information conveyed by the GPS device)? Did the use of this explicit navigational tool guide your attention toward these environmental features to inform your navigation? Or, did it promote an ongoing dependency by continually informing you of your current route relative to the one already prescribed for you? Pre-empting the rhetorical nature of these questions, we ask you to consider now how a learner in sport may miss such information-rich subtleties within a performance environment if ‘navigation’ (i.e., actively engaged problem-solving) is being continually (re)organised for him/her by an external, global source, such as an instructor, trainer, teacher or coach.

#### Wayfinding: a Process Underpinned by Perception-Action Coupling

As skilful wayfinding can be defined through the successful (self)navigation of distances so vast they cannot be directly perceived by an individual from one standpoint, we are drawn to appreciate that it is predicated on Gibson’s [2, 17] perception-action coupling approach to human behaviour. Notably, successful wayfinding requires a deep engagement of an individual with the environment, which supports the capacity to actively self-regulate during performance; that is, to interact with the environment by solving problems, seeking and detecting information, utilising affordances and (re)organising goal-directed actions based upon one’s intentionality and the constraints of the environment [21].

This perspective draws some support from Gibson’s [2] insights on the differentiation of *knowledge of* and *knowledge about* an environment, aligned with the initial ideas of William James [22]. Notably, *knowledge about* one’s environment provides information which allows us to know about some state of affairs, such as knowing that the Melbourne Cricket Ground (MCG) is located in Australia. This information is of most use for a verbal response to a question *about* where the MCG is located. Contrastingly, *knowledge of* one’s environment refers to the skilful perception and action that enables an intended outcome, such as finding your way to the MCG without use of external technological navigational aids. It is the latter of these two knowledge types that requires an embedded understanding of environmental features that enable the achievement of the task goal of skilfully navigating (actively self-regulating through) uncharted fields in a landscape. For this very reason, simply directing or instructing someone how to travel somewhere (using declaratively explicit navigational aids), or more aptly given our paper, how to solve performance-related problems (traditional, prescriptive pedagogical channels of teaching and/or coaching), can never replace the experience of learning by ‘doing’. It is important to note here that we are not contending that wayfinding is simply the process of placing a learner in an environment and letting them ‘find their own way’, a (deliberate or unwitting) distortion of pedagogies aligned to ecological dynamics [23]. Rather, we contend that teaching a learner to wayfind is an embodied and embedded process [10], in which support practitioners work with a learner to deepen his/her *knowledge of* the environment by guiding his/her attention toward its critical features (defined as *wayfinding aids*, [24]) used to inform intentions, perceptual exploration and action.

It is the functionalist experience of ‘doing’ that exploits the notion that there are endless ways to reach the same or highly similar solutions (i.e., destinations) to problems encountered when engaging in wayfinding [25]. For example, actively self-regulating individuals can take many different routes to get to the same destination (e.g. the MCG as discussed) in much the same way that actively self-regulating surfers can score the same points using a variety of cutting manoeuvres during competition, or how actively self-regulating cricket batters can score the same amount of runs using a vastly different array of shots. We contend that in each of these sporting examples, the individuals are demonstrating wayfinding, not in the literal interpretation of traversing through physical regions of a landscape, but in a more metaphoric and dynamic sense, exemplified by a surfer actively exploring the breaking waves to solve emergent performance problems related to the scoring of points by skilfully detecting the critical informational constraints (e.g. movements of the waves, and directions of currents/wind) that shapes the opportunities for action on a surfboard.

Within ecological dynamics, this constant adaptation of actions has been captured through the notion of system degeneracy; a dynamical concept rooted in complexity sciences that describes how the same output can be achieved through the use of structurally different system configurations or elements [5]. When viewed through this dynamical lens, learning how to skilfully wayfind would require a continued sensitivity to the developing context for actions and, in this sense, self-regulation requires one to be open to the invitations for action provided by an environment. The process of wayfinding, predicated on ecological dynamics, can therefore not be facilitated via rote learning, unless the exact constraints of an environment and the intentions of the learner remain near identical from trial to trial, which is rarely, if ever, observed. Nikolai Bernstein [26], the eminent Russian physiologist, understood this point well, arguing that practice should be conceived as a process of “repetition without repetition”, implying that the learning process should not consist of repetition of a movement template. Rather, he proposed that movement training without “repetition without repetition” is… “mere mechanical repetition by rote, a method that has been discredited in pedagogy for some time” ([26], p. 134).

**Fig. 1:**
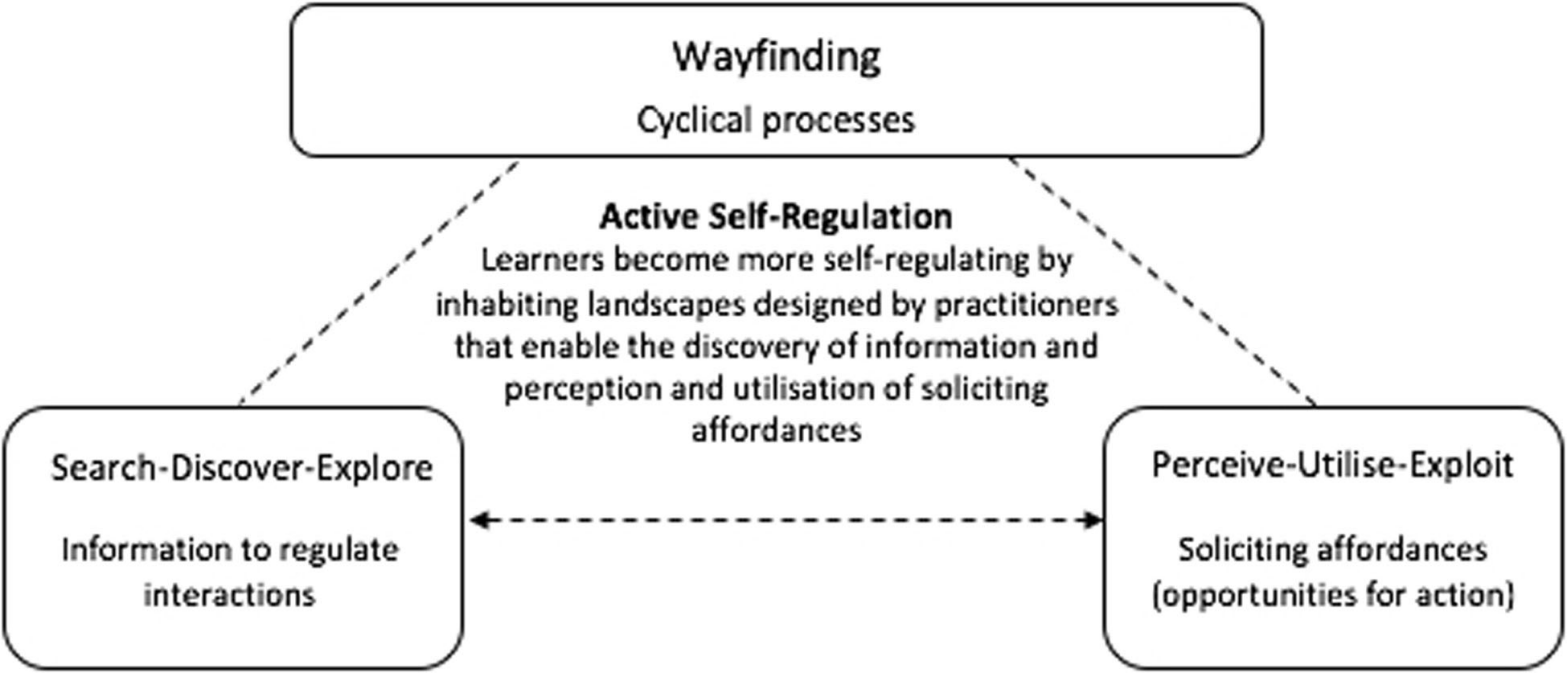
Wayfinding and its cyclical processes. *Note:* As shown in this figure, learners become self-regulating through gaining *knowledge of* the environment by detecting information to utilise affordances available in fields of a performance landscape designed by practitioners

This conceptualisation of practice is aligned with why Gibson [2, 17] conceived wayfinding as the continued process of attuning to (i.e., detecting) information that specifies properties of an environment. However, Gibson did not explicitly relate the process of wayfinding to the utilisation of an environment’s affordances (opportunities for action), which we seek to do here (see Fig. 1). As individuals move through a field in a landscape, their intended actions guide their perceptions and vice versa; thus the process of wayfinding involves the continued, dynamical search and exploration for information that supports the functional adaptation of actions (Fig. 1). Skilful wayfinders are, therefore, individuals who are constantly responsive to the environmental information inviting interactions with available affordances—deepening their *knowledge of* these interactions as they ‘find their way’. When rationalised this way, skill acquisition can be better understood as skill ‘adaptation’ [9].

This dynamic understanding of a skilful wayfinder, as a discoverer of information for actions which make use of available affordances in performance landscapes (Fig. 1), aligns with our re-configuration of the learning process for learners at different performance levels in sport. Appreciating our perspectives of wayfinding predicated on ecological dynamics, practitioners may be drawn to appreciate the need to develop a highly adaptive, emotionally engaged, motivated and self-regulating individual who relies on perceptions and actions to function effectively. This re-conceptualisation of learning has clear implications for support practitioners as learning designers. To develop such self-regulating wayfinders within sport, practitioners would be required to (co)design information-rich ‘landscapes’ that consist of affordances that learners can perceive and learn to utilise toward the achievement of a task goal. Thus, while acknowledging the differences from its literal connotation, we argue that when applied to sport performance, and predicated on an ecological dynamics rationale, wayfinding could be understood as a process of continued attunement to surrounding information that specifies functional properties of an environment, what they afford, realised as a learner ‘navigates’ through emergent problems and challenges designed into the landscape by the sport practitioner.

#### Wayfinding by Means of Affordance Perception and Utilisation

To better understand the affordance concept and its centrality to wayfinding, it is worth considering Gibson’s [2] conceptualisation of an animal’s environment. He argued that an animal’s environment was rich in information, possessing a ‘manifold of opportunities’ for action, referred to as affordances [17]. Affordances have both objective and subjective properties and are specific to an animal through the detection of the information available in the structure of ambient energy arrays [27, 28]. An important consideration of this idea is that the information specifying affordances should be understood as a property of animal-environment interactions [28, 29], with Gibson ([30], p. 411) proposing that “affordances do not cause behaviour but constrain and control it”. For example, resonating with the ideas explored in this paper, Warren [31] asked how individuals moved through environments varying in slopes, inclines and surfaces. He reported that how, or if, stairs are climbed is directly relational to the action capabilities and dimensional properties of an individual [31].

Warren’s [31] findings revealed that affordances have both body (e.g. limb length) and action (e.g. power output) scaled properties, which evolve as an individual’s action capabilities change and develop [27]. This observation indicates that affordances may solicit individuals to act upon them at different time points. Designing practice tasks which solicit individuals to seek and utilise affordances is a major challenge for sport practitioners, ensuring a functional match with a developing learner’s action capabilities (effectivities) [32]. This design challenge implicates the integration of performance analytics and skill adaptation in sport to ensure a tight fit between the developmental status of a learner and the specific constraints of a learning environment [8]. The closeness of fit between a learner’s effectivities and the affordances of the environment is developed through the education of their attention toward detecting relevant information needed to perceive and realise the most inviting, or soliciting, affordances [33].

Current thinking on affordances is that they offer invitations for action, not just opportunities [34]. Specifically, using building designs from architecture in conjunction with phenomenological concepts, Withagen et al. [34] argued that affordances not only provide opportunities for action, but through purposeful design (in architecture), may invite or repel actions. While more empirical work is needed, this proposition emphasises the performer-environment mutuality at the heart of the affordance concept, but also uncovers an interesting question that is of particular relevance to wayfinding; that being, what are the properties of specific affordances that invite their realisation by certain individuals?

While acknowledging the magnitude of this question based on a range of constraints, Withagen et al. [34] did highlight four features that are likely to impact upon affordances’ invitational nature, which in turn, implicates their role in wayfinding; including (i) the action capabilities of the individual (reinforcing the relational properties of information specifying affordances), (ii) evolutionary perspectives related to the survival of a species, (iii) culture and (iv), personal history^1^. Pertinent to the scope of this paper, the idea that affordance invitations are impacted by an individual’s current action capabilities captures the skill of practice design, indicating how an expert practitioner can guide, and when appropriate, nudge a developing wayfinder towards the utilisation of affordances that will support the learner’s regulation of physical, emotional, cognitive and perceptual skills. Thus, while developing wayfinders learn to become more self-regulating, it is an active process that the practitioner supports through carefully designed and soliciting practice landscapes (see Fig. 1). This explanation of human performance now leads us to consider the need for practitioners to view themselves as designers across a landscape of affordances that span a continuum from *generality* to *specificity* of practice contexts if we are to fully appreciate learners as wayfinders, and the learning process as wayfinding when applied to sport and predicated on ecological dynamics.

### Learning in Sport as Wayfinding

#### Practitioners as Designers and Learners as Wayfinders

It is important to appreciate that sports practitioners are designing programmes across multiple timescales of performance, learning and development in an effort to enhance the children and athletes in their care [7]. From a design perspective, one of the greatest challenges for practitioners, regardless of context (physical education to high performance sport), is to develop programmes that are not episodic snapshots, but are interconnected and have continuity across performance, learning and development, thereby supporting physical literacy. The learner and practitioner enter into a collaborative appreciation of wayfinding and a shared ethos of *representative co-design* [36]. In doing so, they move away from a traditional, hierarchical model of the learner-practitioner relationship, characterised by mechanistic perspectives of the learning process, in which the practitioner is at the core of the instructional process, providing the learner with instructions for solving problems, as well as sequentially corrective feedback for continued reproduction and compliance [37, 38]. This traditional approach is synonymous with a navigational device indicating a ‘wrong’ turn that deviates from the prescribed ‘best’ or ‘fastest’ route, which we now appreciate would be more reflective of *transport*, not wayfinding [10].

Sport performance landscapes are continuously evolving. Accommodating for this by prioritising learner-environment interactions, a practitioner can design a practice landscape that invites a learner to explore and exploit available affordances during the learning process. In this sense, there would be no ‘wrong’ turns, just opportunities for learners to continually explore system degeneracy displayed in a variety of potential ways (i.e,. ‘routes’) of solving performance problems within the confines of the landscape designed, being free to settle on a particular solution (i.e., ‘destination’) they feel satisfies their immediate needs and intentions, consonant with their action capabilities and satisfying environmental constraints. An example of such an approach in high performance sport could involve a practitioner designing a practice landscape that encourages particularly difficult or more creative passes between teammates in team sports like rugby union or football, inviting learners to explore and experience *ways* of performing them to penetrate an opponent’s defence. Indeed, while these athletes would not be physically navigating to another practice stadium (as per literal connotations of wayfinding), they are finding their way through different fields or regions within their current performance landscape, exemplified by searching for ways to penetrate an opposition defence, a process shaped by emergent and decaying constraints, some of which are manipulated by a practitioner.

To teach wayfinding, when predicated on ecological dynamics, is to embark on an embodied and embedded process in which a practitioner works to deepen a learner’s *knowledge of* the environment, and in doing so, works across a continuum of affordances of, more or less, specialised performance environments. At the *specialised* end of the continuum, there are fewer, more specific affordances, with this type of practice more suited to the high end of performance sport, where elite athletes spend a large percentage of their time specifying and refining the detection of key information from highly representative performance environments. At the other more *generalised* end of the continuum, there is a more diverse and extensive range of affordances. It is here that participants in physical education (PE) classes will spend the largest percentage of their time “learning to learn how to move” ([39], p. 8). The more generalised the PE programme is, the greater the opportunities for skill adaptation and synergy (re)formation amongst motor system degrees of freedom will be [9]. Experiences of synergy (re)formation will lead to a greater breadth of movement attractors (stable states of coordination) to support functionality. Stated more apparently, greater movement flexibility will enable a child to solve emergent problems in more efficient, creative and adaptable ways, as more opportunities for interaction become available to them. This re-shaped attractor landscape for learners will increase the likelihood that children will become proficient and confident in their own ability to function (perform successfully) across multiple sporting and physical activity environments (i.e., learning to actively self-navigate through a range of diverse problems within a performance landscape). At the more *specialised* end of the continuum, the elite sportsperson will be empowered to create stable and deep attractor wells that will be more resistant to perturbations during competition, yet retaining inherent flexibility.

Sport practitioners can deepen a learner’s *knowledge of* the performance environment and promote wayfinding regardless of where he/she fits on the *specificity-generality* continuum through the use of appropriate teaching styles, such as inquiry-teaching, tactical-games, co-operative learning, discovery and problem-solving [40]. These diverse teaching styles place the learner-environment interaction at the centre of the learning process and will challenge the learner to experiment through performing, adapting and creating movement solutions that best answer his/her individual needs within a given context. Moreover, the learner is learning how to wayfind through problems carefully designed into the activity by the practitioner. The skilled practitioner can enhance this learning experience through the use of targeted questioning [41] that creates an external focus of attention, exploiting self-organisation tendencies for coordination to meet specific task goals [42].

The use of questioning fits eloquently when we conceive the learning process in sport as wayfinding, and interestingly, has been used as a means of educating the skills of humans for centuries [10, 35]. Specifically, in seemingly barren arctic landscapes, skilful wayfinding has been taught through careful questioning that directs individual’s attention toward subtle variances in snow properties and formations that are created by small changes in ambient temperature and the wind’s direction/strength [10, 35]. Even in an uncharted region, it is the detection and utilisation of these information-rich snow formations (situated as affordances from a Gibsonian perspective) taught through the use of questioning, that enables inexperienced hunters to learn to find their way [10, 35]. We argue that this case example is synonymous with a practitioner in sport using questioning to educate the attention of learners toward the detection of, for example, a bowler’s finger placement on a ball in cricket, or the positioning of a defender during a game in hockey. The detection of this information could guide the athletes’ (re)organisation of action, using it to find their way through emergent and novel performance problems encountered, conceptualised as uncharted fields of the performance landscape.

It is important to note here that questioning needs to be answered not by verbal responses (echoing Gibson’s concept of *knowledge about* an environment) but rather by opportunities for the (re)organisation of actions (synergy re-formation). In such a way, educators and/or mentors are not problem-solving (i.e., navigating) for learners (acting like a personal GPS device), but are assisting them to wayfind by deepening *knowledge of* their environmental niche through guiding their attention toward soliciting affordances that are rich in meaning. Stated differently, the use of questioning could serve as a basis to educate the attention of the learner toward the perception and utilisation of opportunities for action that support wayfinding (for a detailed insight into the education of attention, see [43]). Thus, practitioners in sport could be viewed as *wayshowers* that guide or educate the search of wayfinders, not by telling them what to see, but by showing them where to look through carefully designed performance landscapes.

In the remaining sections of this paper, we embed learning in sport as wayfinding by offering examples as to how practitioners at multiple stages of athlete development could design environments that allow learners to actively self-navigate uncharted, performance-related problems (situated as fields in a landscape). Specifically, we offer two examples from either end of the landscape of affordance continuum in which practitioners have enacted learning designs guided by the concept of wayfinding, namely, within a PE curriculum (generality), and high performance sport setting (*specificity*). However, prior to elaborating on these examples, we re-iterate that our applications of wayfinding, predicated on ecological dynamics, are not literal in the sense that an individual must navigate between physical regions in a landscape by moving through different vistas. Rather, we situate wayfinding as the process of learning to search for, and detect, information in an environment that individuals learn to exploit for solving (i.e., ‘navigating’) performance-related problems, situated as fields (regions) within an evolving landscape.

#### Example 1 —Generality: Teachers as Designers and Students as Wayfinders in Early Years Physical Education

Within a pre-school and early primary school PE setting, we suggest that the majority of time should be spent at the generality end of the continuum, with less time being spent in specificity of practice—for example, learning how to get changed and prepared for PE and lining up at the classroom ready to go into the play space landscape (i.e., hall). Once the children enter the play space landscape, the teacher spends his/her time at the generality end of the spectrum, where a carefully designed curriculum could offer enriched and personally challenging problems (fields to navigate through) to support the development of temporal and rhythmic movements (through dance, for example); postural control and balance skills (through tumbling, jumping, controlled falling and moving in gravity-defying ways through gymnastics, for example); as well as hand-eye coordination (through ball games, for example). The culmination of such an enrichment programme will see children develop their movement signature whilst developing major components of performance; stability, flexibility, rhythm, agility and power [44, 45]. These progressive changes to a child’s action capabilities will likely afford them ‘new’ opportunities for interaction that support wayfinding behaviours. For example, a child who develops more pronounced hand-eye coordinative skills may be afforded more opportunities to wayfind through emergent problems in ball games that require interceptive actions [39].

To support wayfinding, the teacher could use analogies and questions on a common theme to encourage problem-solving and exploration by the child, rather than the child being told by the teacher exactly what to do. For example, a lesson based on wildlife could see children becoming a snake, affording them opportunities to move their bodies close to the floor and over and under equipment. This would enable them to perceive their landscape from a different vantagepoint. Further, *knowledge of* other animals could be explored, allowing contrasting opportunities for movement that coincide with changing perceptions of the play space landscape. For example, a monkey could be used as an analogy to help children wayfind through ‘high’ fields of their landscapes, being encouraged to ‘navigate’ through these fields of their landscapes by balancing on, swinging over, hanging to and landing between different obstacles using their hands, feet, arms and legs. This landscape of affordances would be found toward the generality end of the continuum, as it is non-sport specific and consists of very few specific constraints on the task, allowing the children to demonstrate a wide variety of functional movements within their own action capabilities. These more general movement experiences provide opportunities for children to learn to move [39] by engaging in continuous synergy (re)formation in order to utilise a rich range of affordances available in the diverse fields of the landscape.

Co-designed mini-games within the lessons can also promote wayfinding when coupled with teaching methods such as analogies and questions. For example, children might create an imaginative game that involves a snake moving treasure (beanbags) across a stream (their gym mat). The teacher can add informational constraints to the game to nudge skill development, such as ‘the treasure cannot get wet’ (challenging children to keep the beanbag off the mat/floor), and to move each piece of treasure, the children must roll across the water in a different way otherwise the monkey might spot them. These types of activities create an external focus of attention, whilst problem-solving, requiring imaginative (re)organisation of functional movement solutions, remains at the heart of the activity. With this approach, there is no external prescription of ‘correct or optimal’ movement solutions (reflective of a prescriptive way of solving the problem). Rather, the teacher seeks to continuously infuse perturbations within the learning process through destabilising a learnt skill by altering task constraints, or changing the task goal (reshaping the fields within the landscape). If done well, this teaching approach will result in the perception and utilisation of ‘new’ affordances and skill adaptations by learners as they experience novel and uncharted fields of the performance landscape. While these manipulations are at the teachers’ discretion, it is important that they understand that it is acceptable for children to display different movement solutions for the same task (exemplifying different ‘routes’ to the same ‘destination’) and that regression in a skill is inevitable when altering constraints (such as changes in equipment). The teacher should also keep in mind that, as long as the skill is functional and achieves the outcome of the lesson (satisfies children’s intentions), then it is an acceptable solution for the purposes of wayfinding.

#### Example 2 —Specificity: Coaches as Designers and Athletes as Wayfinders in High Performance Sport

In contrast to the above, for specialising athletes in high performance sport, we would suggest that the majority of the time is spent at the specificity end of the continuum, with less at the generality end. For example, more generalised practice designs may afford athletes opportunities to explore divergent and creative ways to solve problems (navigating through vastly different fields of the performance landscape) in safe, but still uncertain, contexts. At the specificity end of the continuum, coaches would design, and expose athletes to, performance landscapes that are rich in meaning (i.e., are representative of the constraints experienced in competition), allowing them to fine tune their capacity to detect information that specifies the more subtle properties of affordances of use to solve specific performance-related problems. To achieve this design goal and deepen an athlete’s knowledge of a specific field within a performance landscape, coaches could use a range of pedagogical channels such as constraints’ manipulation and carefully targeted questioning (as was discussed earlier and used within the previous example). When used appropriately, both approaches will direct athletes to be more actively self-regulating in performance, that is, attending to, detecting, utilising and exploiting information for the most soliciting affordances in their landscapes (as depicted in Fig. 1).

In this example, a cricket coach is designing a practice task (performance landscape) in which a batter, and subsequent batting team, has a defined number of runs to score in a specified number of deliveries. This nuanced challenge may be situated in a vignette, late in a T20 (20 over) game, where the fielding team is trying to prevent runs being scored. While the batter is not physically moving through different regions in a landscape in a literal sense, such a task design will still (metaphorically) enable wayfinding, as the batter (and batting team) will have to navigate through dynamic and likely unexplored fields (i.e., problems) within the performance landscape in an effort to solve the global problem (i.e., winning the game by outscoring the opposition within the defined number of balls). This is a highly dynamic landscape, as constraints such as missed shots, wickets, the use of power plays by the fielding team, ball placement, and changing weather and light conditions would continually shape (or constrain) how the batters score runs. Simply, each ball faced is a ‘new’ field they are yet to explore. Further, this landscape design immediately invites the batter to search for information that affords them with *ways* to continuously score runs, under intense defensive fielding pressure.

To help guide the batters search, the coach could manipulate the field by placing more fielders in the locations that favour the batter’s known, preferred hitting zones (a strategy mimicking an approach by the fielding team in an actual game). This landscape manipulation will challenge the batter to recognise where the fielders are located and to perform a shot that avoids their interactions in order to score runs. More directly, the changing location of the fielders will likely change the batter’s perceptions of how to score runs, (figuratively) changing their vantage point of the performance landscape. To further guide the recognition of fielder’s position, the coach could question the batter about *where* the fielders are placed, *where* he/she perceives the bowler could deliver the ball based on the field, and *how* he/she could adapt a shot to exploit the gaps located in the specific set field. In each question, the coach is attempting to deepen the *knowledge* *of* the batter by helping him/her identify the most soliciting affordances within their field that enable the achievement of the task goal (i.e., teaching them to actively self-regulate their perceptions, emotions and actions). Coaches do not need to tell the batter what to see, but rather facilitate wayfinding by him/her appreciating where to look. This is because no explicit instruction has been given to the batter about what shot to perform, with (s)he being free to explore this action within the different fields (i.e., problems) encountered. For example, a missed shot may invite the batter to explore a riskier shot on the next ball in an attempt to score more runs, which demonstrates the constantly dynamic fields the batter must learn to find their way through. The learning process is, therefore, constituted by the coach designing the specific practice landscape that consists of dynamic problems (uncharted fields), guiding the search of the batter using a range of pedagogical channels (wayshowing), and then allowing him/her to wayfind through such problems by detecting information and utilising soliciting affordances based on his/her action capabilities.

#### Summary of Both Examples

Despite being at different ends of the *specificity-generality* continuum, sports practitioners in both examples adopted similar principles of affordance design and provision of opportunities for individuals to (re)form synergies. Therefore, when the learning process in sport is framed through wayfinding, practitioners, inhabiting all locations on the skill continuum, need not map pre-determined ‘routes’ in some cartographic manner for learners. Nor do they need to place the learner in a performance landscape with the open-ended and non-guiding instruction of ‘figure it out on your own’. Rather, in both examples, enriched performance landscapes, situated in meaning to the learners, were designed, with the practitioners encouraging the learners to navigate through diverse problems (i.e., fields of the landscape) by learning to actively self-regulate their perceptions, actions, cognitions and emotions through the use of different pedagogical channels. By situating the learners as wayfinders, and the subsequent learning process in sport as wayfinding (irrespective of which end of the specificity-generality continuum the practitioner is inhabiting), the practitioners in both examples were encouraged to design activities that invited learner interaction. With this approach, learners can be empowered to perceive affordances of use for navigating through uncharted fields (i.e., problems) within landscapes by continuously re-organising their motor system degrees of freedom. We contend that from this perspective, learners can never be ‘lost’ within their environmental niche, as they have embarked upon a lifelong journey of continually deepening their connectedness to the environment. Moreover, they are learning how to skilfully adapt stable movement traces (synergies formed by movement system components) through the detection of information that specifies opportunities for action that enable the achievement of a task goal, whatever that task goal may be along a self-chosen ‘route’.

### Concluding Remarks

As highlighted by M.R. O’Connor in the opening quotation, skilful wayfinding is an embodied and embedded process where individuals learn to self-regulate actions by connecting to the environment. It is through this connection that individuals learn to detect and experience its many subtleties, understanding how these environmental subtleties can be used to successfully navigate through uncharted fields within a landscape. More simply, the ‘journey’ is what leads to the requisite growth of knowledge wayfinders exploit to find their way. The intention of this commentary was to introduce this concept to sport practitioners and applied scientists, demonstrating the congruence between wayfinding and how we perceive learners and learning processes through an ecological dynamics lens. Indeed, our propositions were more metaphoric in nature, as we conceptualised regions or fields individuals learn to navigate through as performance problems, and the broader landscape as a sporting task or activity. Thus, our perspectives of wayfinding were more attuned to the underlying processes of how individuals learn to navigate dynamic performance environments—linking these processes to how we understand learners in sport through an ecological dynamics lens. By re-configuring our understanding of learners in sport in this way, we contend that practitioners could better enact learning designs that deepen a learner’s connection with a niche in a performance environment. In this sense, students and athletes learn to continually self-regulate unencountered performance-related problems in much the same way humans learned to navigate the world, and their place within it, without the use of advanced technological aids—as, whether in navigation, sport, or life “…wayfinding isn’t knowing *before* we go, but, knowing *as* we go” [10].

## Data Availability

Not applicable

## Footnotes

1 At this point, we would like to nudge the reader toward M.R. O’Connor’s book on wayfinding [35] and Ingold’s book on the perception of the environment [10]. Within them, they will find detailed and fascinating insights into how each of these features shaped the navigational skills of ancient civilisations engaging in masterful wayfinding.
